# Lens thickness and associated ocular biometric factors among cataract patients in Shanghai

**DOI:** 10.1186/s40662-021-00245-3

**Published:** 2021-05-31

**Authors:** Jiaqi Meng, Ling Wei, Wenwen He, Jiao Qi, Yi Lu, Xiangjia Zhu

**Affiliations:** 1 Eye Institute and Department of Ophthalmology, Eye & ENT Hospital, Fudan University, 83 Fenyang Road, Xuhui District, Shanghai, 200031 China; 2 Key Laboratory of Myopia, Ministry of Health, Shanghai, China; 3 Key Laboratory of Visual Impairment and Restoration, Shanghai, China; 4 Key NHC key Laboratory of Myopia (Fudan University), Shanghai, China; 5 Laboratory of Myopia, Chinese Academy of Medical Sciences, Shanghai, China

**Keywords:** Lens thickness, Cataract, Ocular biometry, Axial length

## Abstract

**Background:**

To evaluate the distribution of lens thickness (LT) and its associations with other ocular biometric factors among cataract patients in Shanghai.

**Methods:**

Twenty-four thousand thirteen eyes from 24,013 cataract patients were retrospectively included. Ocular biometric factors including LT, central corneal thickness (CCT), anterior chamber depth (ACD), white-to-white (WTW) distance, anterior corneal curvature, and axial length (AL) were obtained using the IOLMaster700. The associations between LT and general or ocular factors were assessed.

**Results:**

The mean age was 62.5 ± 13.6 years and 56.1% were female. The mean LT was 4.51 ± 0.46 mm. The LT was greater in older patients (*P* < 0.001). LT was positively correlated with CCT, while negatively correlated with ACD, WTW, and anterior corneal curvature (*P* < 0.001). Multivariate analysis revealed that increased LT was associated with older age, male gender, thicker CCT, shallower ACD, larger WTW, and flatter anterior corneal curvature (*P* < 0.001). LT changed with a variable behavior according to AL. In short eyes LT increased as AL increased, then decreased with longer AL in normal eyes and moderate myopic eyes, but increased again as AL increased in highly myopic eyes. Thickest LT was found in the 20.01–22 mm AL group. The correlation between LT and other biometric factors remained significant when stratified by ALs.

**Conclusions:**

In a large Chinese cataractous population, we found that the thicker lens may be associated with older age, male gender, thicker CCT, shallower ACD, larger WTW, and flatter anterior corneal curvature. As AL increased, the change of LT was nonlinear, with the thickest lens seen in the 20–22 mm AL group.

## Introduction

Currently, cataract is one of the leading causes of visual impairment worldwide [1]. With the advancement of cataract surgery and intraocular lens (IOL) design, the accurate biometric measurement has become a crucial factor in surgical planning [2]. Lens thickness (LT) is one of the important parameters in ocular biometry. Not only is it a necessary variable used in fourth-generation IOL formulas [3–5], but also its associations with other ocular parameters may further affect actual lens position after surgery [6]. Meanwhile, during cataract development, morphological changes of the lens may also occur along with decreased transparency [7, 8]. Therefore, the observation of LT among cataract patients is of great importance from both clinical and pathophysiological perspectives.

Recently, accurate measurement of LT in cataractous eyes has become available due to the advancement of optical biometry [9–11]. Based on the optical low-coherence reflectometry (OLCR), an increased trend of LT with cataract formation was identified [9], while based on swept source optical coherence tomography (SS-OCT), Henriquez et al. found that the increase of LT was independent of lens density in mild to moderate cataract [10]. Moreover, OLCR-based LT contributes to lower refractive error compared with age-derived LT [11].

Due to changes in anatomical structures of eyeballs with different axial lengths (AL) [12], the characteristics of ocular biometry may also show variations. Longer AL is associated with higher central cornea thickness (CCT), higher corneal radius, larger corneal diameter and deeper anterior chamber depth (ACD) in normal eyes [13–15], but these correlations may be not present in eyes with extreme long or short AL [16, 17]. Vega et al. found that the effect of LT on the accuracy of the Barrett Universal II formula was the greatest in eyes with AL ≤ 22 mm but less in eyes with AL ≥ 26 mm [18]. Even among patients with normal ALs (22–26 mm), a higher prediction error may be seen with an extreme LT, especially for the Haigis and Hill-RBF V2.0 formulas [19]. Thus, investigating the distribution of LT against AL is required. Previously, reports on LT were seen in countries such as Peru, the United States, Iran and Portugal [10, 13, 20, 21]. A thinner lens was reported in the Iranian or Portuguese population than the Peruvian or American study, indicating a potential racial difference in LT. With higher prevalence of myopia and high myopia in Asia, the lens geometry of Asian eyes may also be different from that of Caucasian eyes. However, no reports on LT have been published that include a large sample of Asian cataractous eyes.

Thus, in this study, based on a large sample, we aimed to investigate the distribution and associated ocular biometric factors of LT in cataractous eyes in Shanghai, and also to focus on the influence of AL on LT.

## Methods

The protocols for this retrospective observational study were approved by the Institutional Review Board of the Eye and Ear, Nose, Throat (EENT) Hospital of Fudan University, Shanghai, China (ID: 2014055). The study adhered to the tenets of the Declaration of Helsinki. Written informed consent for the use of clinical data was routinely obtained from each patient before cataract surgery.

### Patients

Ocular biometry data of patients who had cataract surgery between March 2018 and March 2020 were reviewed. Patients aged 18 years or older were included. We excluded eyes with marked corneal abnormalities that would affect measurement (e.g., dense corneal scars, keratoconus, and irregular astigmatism), lens dislocations, active ocular inflammation, and previous trauma or intraocular surgery. When both eyes of a patient met the criteria, we randomly selected one eye from each patient for analysis using the random number table. In brief, the left eye was included if the patient was given an odd random number, otherwise the right eye was included. For patients in whom only one eye met the criteria, that eye was included. Finally, a total of 24,013 eyes of 24,013 patients were available for analysis.

### Ocular biometric measurements

For each eye, ocular biometric factors, including LT, CCT, ACD, white-to-white (WTW) distance, anterior corneal curvature and AL, were measured using a swept-source optical coherence tomography (OCT) based biometer (IOLMaster700, version 1.80; Carl Zeiss Meditec, Jena, Germany). For this device, axial measurements, including LT, CCT and ACD are based on swept-source frequency-domain optical coherence tomography with a 44 mm scan depth and 22 mm tissue resolution. LT was defined as the distance between the anterior and posterior poles of the crystalline lens. CCT was defined as the distance between the corneal epithelium and endothelium. ACD was defined as the distance between the corneal endothelium and anterior crystalline lens surface. The device also uses a scleral and iris image for WTW measurement, and the reflected light spots on the corneal surface for anterior corneal curvature measurement. All measurements were routinely performed by experienced technical staff in a single examination lane within the same institution using a single IOLMaster700 device. During each measurement, the technical staff visually checked the eye geometry and axis of the measurements on the scan image of the entire eye, and ensured the correct fixation by the patient on the foveal scan. For each measurement, the device calculates the standard deviation (SD) for LT, ACD and AL and warned of poor-quality results if the SD for LT > 0.038 mm, for ACD > 0.021 mm and for AL > 0.027 mm [22]. Measurements with poor-quality results were deleted and remeasured until reproducible readings were obtained. Data were stored in a spreadsheet before being input into SPSS software for analysis.

In this study, the eyes were further divided into eight groups according to the AL (AL ≤ 20 mm, 20.01–22 mm, 22.01–24.5 mm, 24.51–26 mm, 26.01–28 mm, 28.01–30 mm, 30.01–35 mm, and > 35 mm), Then, eyes with AL ≤ 22 mm were defined as short eyes, those with AL ranging from 22.01 to 24.5 mm were defined as normal eyes, those with AL ranging from 24.51 to 26 mm were defined as moderate myopic eyes, and those with AL > 26 mm were defined as highly myopic eyes.

### Statistical analyses

Continuous data were presented as means ± standard deviations and categorical data were presented as proportions (%). Kolmogorov-Smirnov test was used to assess normality. Mann-Whitney *U* test or Kruskal-Wallis test was used to compare continuous data between groups or among three or more groups. Analysis of covariance (ANCOVA) was used to compare the LT between groups or among three or more groups after adjustment for age. The Pearson correlation analysis was used to assess the associations between LT and general or other biometric parameters. The stepwise backwards multiple linear regression analysis was performed with LT as the dependent variable and general or other ocular biometric factors as independent variables. The odds-ratio (OR) was calculated using a binary logistic regression model, in which a thick lens (LT > 4.51 mm) was defined as 1 and a thin lens (LT ≤ 4.51 mm) was defined as 0. All statistical analyses were performed with SPSS version 22 (SPSS, Chicago, IL, USA). Two-sided *P* values < 0.05 were considered statistically significant for all analyses.

## Results

### Characteristics

Table 1 lists the general and ocular characteristics of the study population. Of the included 24,013 patients, the mean age was 62.5 ± 13.6 years (Median, 64.0 years; Range, 18.0–101.0 years), and 13,450 (56.1%) were female. The mean AL was 24.71 ± 2.81 mm; Range, 14.07–37.96 mm.

**Table 1:** Study population characteristics

	Total (*N* = 24,013)
Age (years)	62.5 ± 13.6
Sex (male/female)	10,563/13450
Eye laterality (right/left)	11,843/12170
CCT (mm)	0.55 ± 0.04
ACD (mm)	2.52 ± 0.48
WTW (mm)	11.70 ± 0.46
Anterior corneal curvature (mm)	7.69 ± 0.28
AL (mm)	24.71 ± 2.81

*CCT* central corneal thickness; *ACD* anterior chamber depth; *WTW* white-to-white; *AL* axial length

**Fig. 1:**
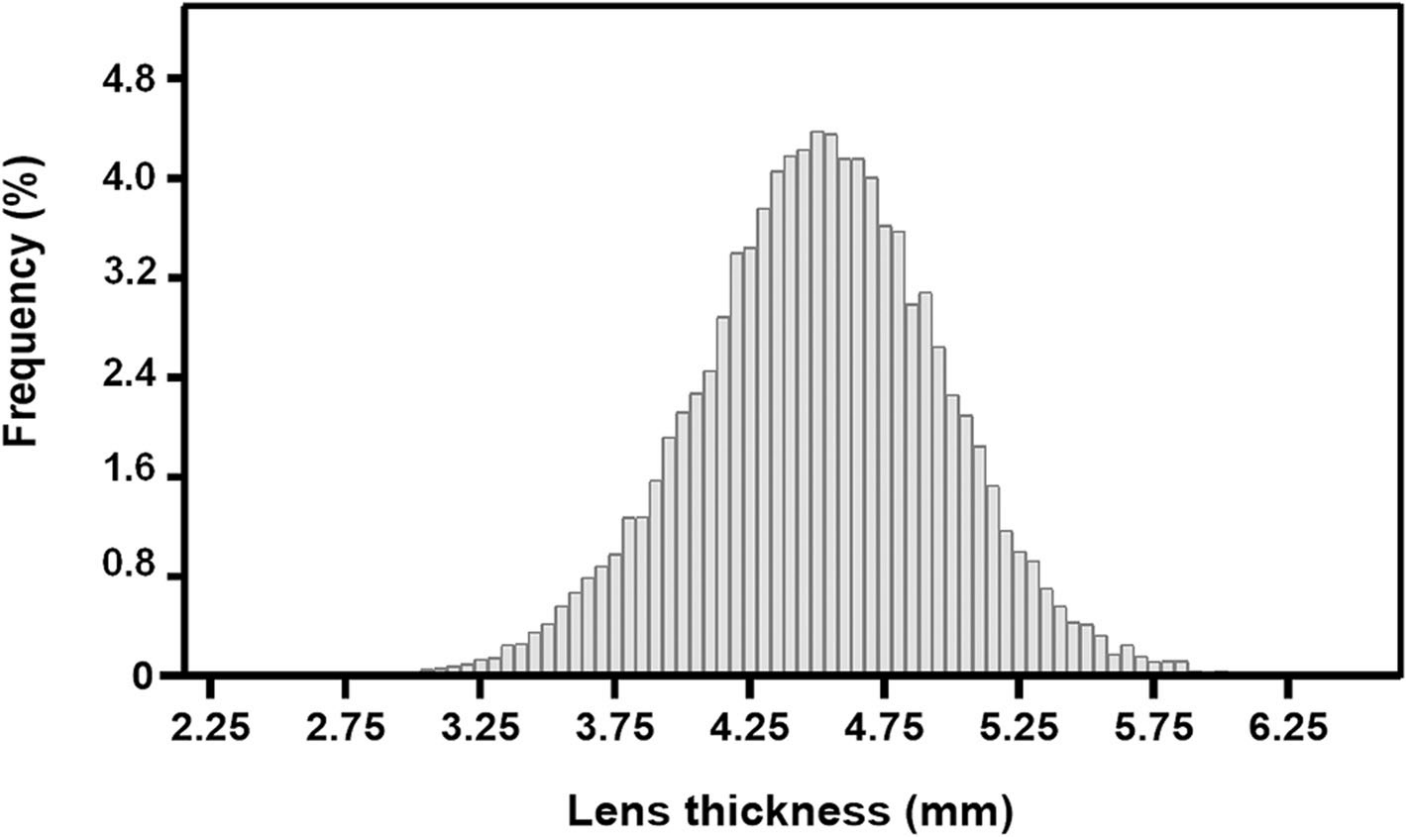
Frequency distribution of lens thickness among the study population

Figure 1 shows the frequency distribution of LT among the study population, with a mean value of 4.51 ± 0.46 mm (Median, 4.51 mm; Range, 2.49–6.36 mm).

### Comparisons of LT according to age and gender

LT stratified by age and gender is presented in Table 2. When LT was stratified by age, in either men or women, LT was significantly greater in older patients (Kruskal-Wallis test, *P* < 0.001). When stratified by gender, LT was generally greater in men than in women (ANCOVA, *P* < 0.001). Of note, in patients aged 60–69 years and 70–79 years, LT was significantly greater in men than in women (Mann-Whitney *U* test, both *P* < 0.05), while no differences were found between men and women in the other age groups (all *P* > 0.05).

**Table 2:** Lens thickness stratified by age and gender

Age (years)	Men			Women			*P* value
	N (%)	Lens thickness (mm)		N (%)	Lens thickness (mm)		
		Mean ± SD	95% CI		Mean ± SD	95% CI	
< 30^a^	386 (3.7%)	3.70 ± 0.39	[3.66, 3.74]	243 (1.8%)	3.72 ± 0.45	[3.66, 3.78]	0.576
30–39^a^	614 (5.8%)	4.00 ± 0.42	[3.97, 4.03]	395 (2.9%)	4.01 ± 0.46	[3.97, 4.06]	0.584
40–49^a^	1241 (11.7%)	4.26 ± 0.38	[4.24, 4.29]	910 (6.8%)	4.30 ± 0.46	[4.27, 4.33]	0.152
50–59^a^	2095 (19.8%)	4.44 ± 0.40	[4.42, 4.46]	2510 (18.7%)	4.42 ± 0.41	[4.40, 4.43]	0.091
60–69^a^	3066 (29.0%)	4.60 ± 0.41	[4.58, 4.61]	5047 (37.5%)	4.55 ± 0.41	[4.54, 4.56]	< 0.001
70–79^a^	2264 (21.4%)	4.67 ± 0.42	[4.65, 4.69]	3213 (23.9%)	4.64 ± 0.42	[4.63, 4.66]	0.012
≥ 80^a^	897 (8.5%)	4.79 ± 0.41	[4.76, 4.82]	1132 (8.4%)	4.76 ± 0.41	[4.74, 4.79]	0.105
Total^b^	10,563 (100%)	4.52 ± 0.47	[4.51, 4.53]	13,450 (100%)	4.50 ± 0.46	[4.49, 4.51]	< 0.001

*SD* standard deviation; *CI* confidence interval

^a^ Mann-Whitney *U* test

^b^ Analysis of covariance after adjusting for age

### Associations between LT and general or ocular biometric factors

**Fig. 2:**
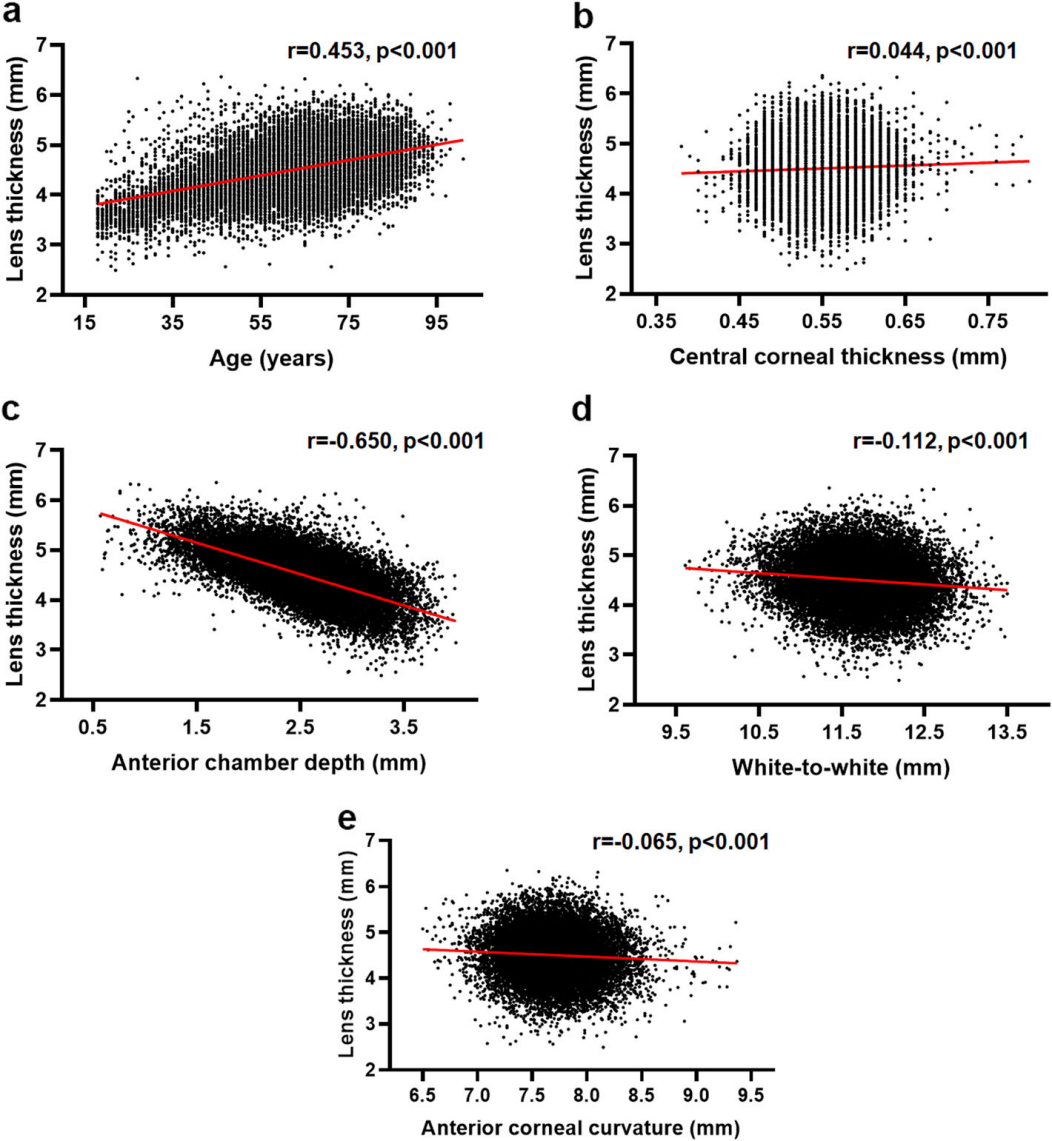
The correlation between lens thickness and age and ocular biometric factors. Scatter plots showing the correlation between lens thickness and age (**a**), central corneal thickness (**b**), anterior chamber depth (**c**), white-to-white distance (**d**), and anterior corneal curvature (**e**) in cataract patients. The red straight line refers to the corresponding equation resulting from the linear regression analyses. Multivariate analysis showed that the thicker lens was associated with older age (β = 0.295, *P* < 0.001), male gender (β = 0.103, *P* < 0.001), thicker central corneal thickness (β = 0.058, *P* < 0.001), shallower anterior chamber depth (β = − 0.748, *P* < 0.001), larger white-to-white distance (β = 0.204, *P* < 0.001), and flatter anterior corneal curvature (β = − 0.113, *P* < 0.001)

**Fig. 3:**
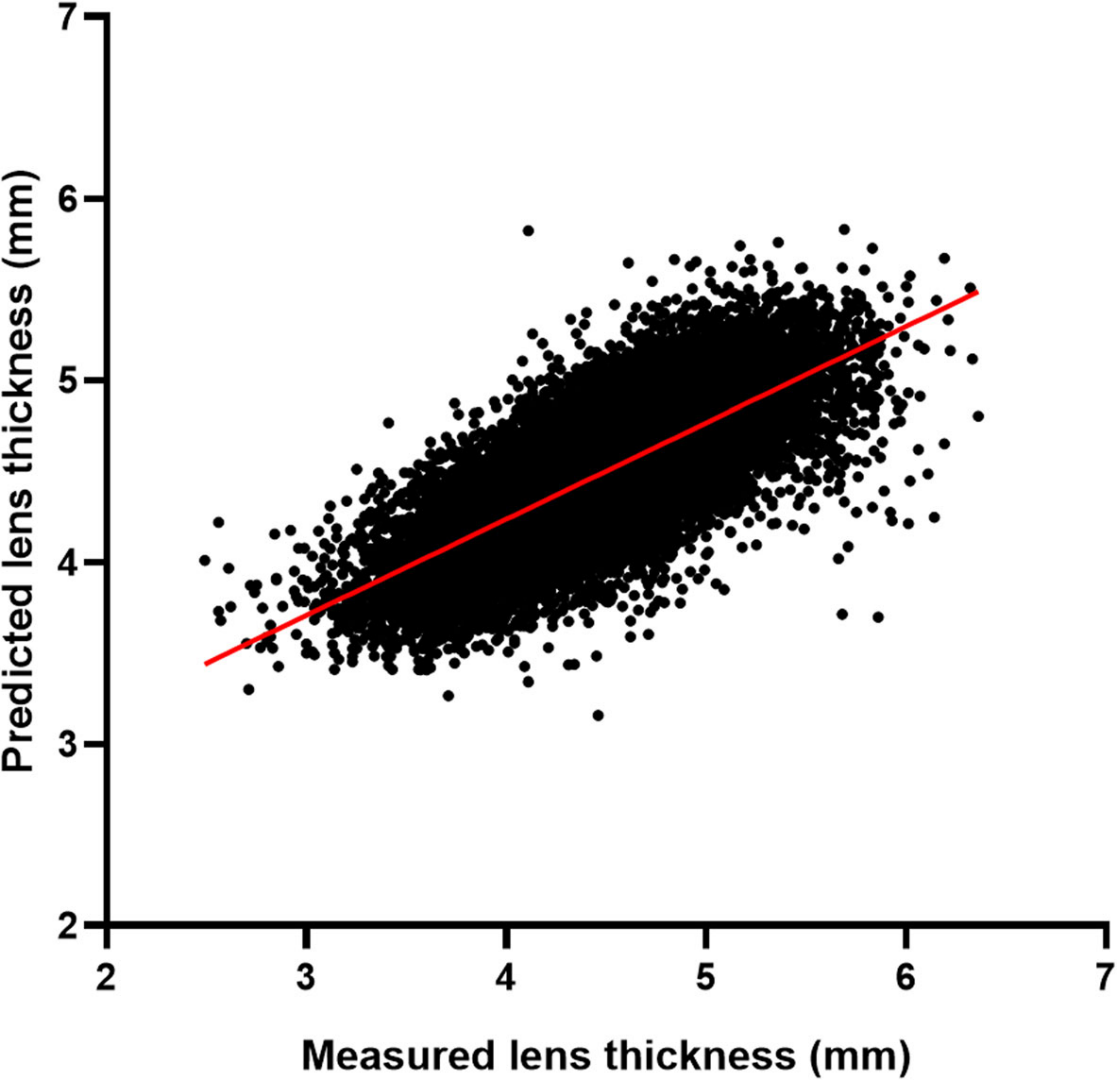
Predicted lens thickness versus preoperative lens thickness measured by the IOLMaster700 in 24,013 Asian cataractous eyes. The predicted lens thickness was calculated using a regression model including age, axial length, anterior chamber depth. The correlation coefficient was 0.73 (*P* < 0.001)

Univariate analysis revealed that LT was positively correlated with age and CCT, while negatively correlated with ACD, WTW, and anterior corneal curvature (Pearson correlation analysis; all *P* < 0.001; Fig. 2). In addition, based on a regression model using AL, ACD and age for LT, the predicted LT versus the LT measured by the IOLMaster700 was plotted in Fig. 3 (predicted LT = 0.226*AL − 0.664*ACD + 0.280*age). This model yielded an adjusted R^2^ of 0.53. The LT measured by the IOLMaster700 could be predicted with a correlation coefficient of 0.73 (*P* < 0.001), and 89.2% of the cases had a prediction error within 0.50 mm of the target.

Binary logistic regression model using general and ocular biometric factors for LT revealed that a thick lens was more likely to be seen in cases of older age (OR, 1.058; 95%CI, 1.054–1.061; *P* < 0.001), male gender (OR, 1.763; 95% CI, 1.643–1.893; *P* < 0.001), thicker CCT (OR, 1.326; 95% CI, 1.241–1.417; *P* < 0.001), shallower ACD (OR, 0.013; 95% CI, 0.012–0.015; *P* < 0.001), larger WTW (OR, 3.362; 95% CI, 3.067–3.685; *P* < 0.001), flatter anterior corneal curvature (OR, 0.334; 95% CI, 0.290–0.385; *P* < 0.001) or longer AL (OR, 1.259; 95% CI, 1.242–1.277; *P* < 0.001).

### Distribution and association of LT in different AL groups

The percentages of short, normal, moderate myopic and highly myopic eyes were 6.2% (1493 eyes), 57.5% (13,801 eyes), 13.0% (3132 eyes) and 23.3% (5587 eyes), respectively. Table 3 shows the comparisons of LT according to AL. After adjustment for age and gender, LT was greatest in the 20.01–22 mm AL group and was smallest in the 26.01–28 mm AL group (*P* < 0.001). As for highly myopic eyes, LT increased gradually with the increased AL, and when the AL was greater than 35 mm, it reached a peak level of 4.72 ± 0.47 mm.

**Table 3:** Comparison of lens thickness according to axial length

AL (mm)	N (%)	LT (mm)	
		Mean ± SD	95% CI
≤ 20	159 (0.7%)	4.62 ± 0.49	[4.54, 4.69]
20.01–22	1334 (5.6%)	4.75 ± 0.45	[4.73, 4.78]
22.01–24.5	13,776 (57.4%)	4.55 ± 0.45	[4.54, 4.56]
24.51–26	3157 (13.1%)	4.39 ± 0.47	[4.38, 4.41]
26.01–28	2435 (10.1%)	4.33 ± 0.45	[4.31, 4.35]
28.01–30	1463 (6.1%)	4.38 ± 0.45	[4.35, 4.40]
30.01–35	1608 (6.7%)	4.53 ± 0.43	[4.51, 4.55]
> 35	81 (0.3%)	4.72 ± 0.47	[4.62, 4.83]
*P* value*		< 0.001	

*AL* axial length; *LT* lens thickness; *CI* confidence interval

* Multivariate analysis after adjustment for age and gender

**Fig. 4:**
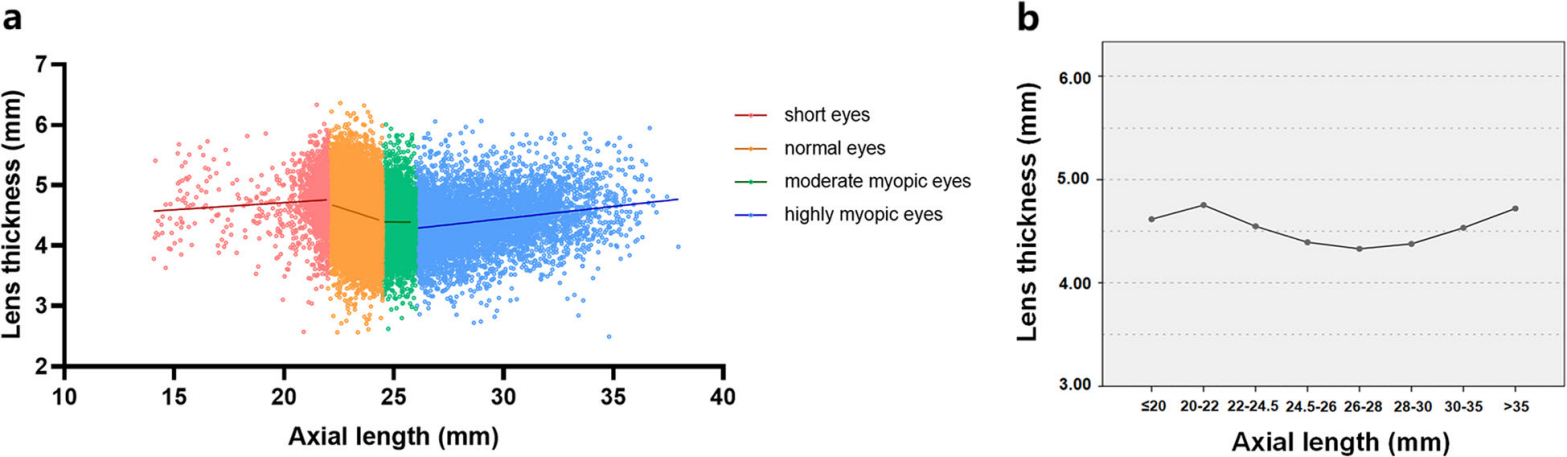
Distribution of lens thickness in dependence of the axial length. **a** Scatter plot showing the LT against AL among cataract patients. In short eyes, the equation resulting from the linear regression analysis (*red line*) was: LT (mm) = 0.024 AL (mm) + 4.241 mm (*r* = 0.076, *P* = 0.004). In normal eyes, the equation resulting from the linear regression analysis (*orange line*) was: LT (mm) = − 0.117 AL (mm) + 7.281 mm (*r* = − 0.166, *P* < 0.001). In moderate myopic eyes, no significant correlation was found between LT and AL (*green dotted line*; *r* = − 0.006, *P* = 0.760). In highly myopic eyes, the equation resulting from the linear regression analysis (*blue line*) was: LT (mm) = 0.040 AL (mm) + 3.235 mm (*r* = 0.207, *P* < 0.001). **b** Histogram showing the comparisons of LT in different AL groups after adjusting for age and gender. AL, axial length; LT, lens thickness

The scattergram of LT against AL showed poor linearity of the fitting curve (Fig. 4a). In both short and highly myopic eyes, LT was positively correlated with AL (Pearson correlation analysis, both *P* < 0.001), while in normal eyes, LT was negatively correlated with AL (*P* < 0.001). No significant correlation was identified between LT and AL in moderate myopic eyes (*P* > 0.05). From Fig. 4b, in short eyes, LT increased as AL increased, then decreased with longer AL in the normal eyes and moderate myopic eyes, and finally LT again increased as AL increased in highly myopic eyes.

In terms of the associations between LT and other ocular biometric factors (Table 4), multivariate analysis showed that greater LT was associated with thicker CCT, shallower ACD, larger WTW, and flatter anterior corneal curvature within each AL group (all *P* < 0.05), after adjustment for age, sex, and eye laterality (right/left).

**Table 4:** Associations between lens thickness and other ocular biometric factors in eyes with different axial lengths

	β	*P* value*
Short eyes		
CCT (mm)	0.078	< 0.001
ACD (mm)	−0.680	< 0.001
WTW (mm)	0.192	< 0.001
Anterior corneal curvature (mm)	−0.067	0.001
Normal eyes		
CCT (mm)	0.051	< 0.001
ACD (mm)	−0.690	< 0.001
WTW (mm)	0.204	< 0.001
Anterior corneal curvature (mm)	−0.079	< 0.001
Moderate myopic eyes		
CCT (mm)	0.059	< 0.001
ACD (mm)	−0.601	< 0.001
WTW (mm)	0.190	< 0.001
Anterior corneal curvature (mm)	−0.146	< 0.001
Highly myopic eyes		
CCT (mm)	0.045	< 0.001
ACD (mm)	−0.579	< 0.001
WTW (mm)	0.193	< 0.001
Anterior corneal curvature (mm)	−0.112	< 0.001

*CCT* central corneal thickness; *ACD* anterior chamber depth; *WTW* white-to-white. β regression coefficient

* Multivariate analysis after adjusting for age, gender, and eye laterality (right/left)

## Discussion

In the last two decades, given the more predictable refractive results of modern phacoemulsification, surgeons have given increasing importance to the use of optical biometers, which have proven to be accurate and quite comparable in many of their measurements [23, 24]. This technological support, together with developments in IOL platforms, has allowed an important growth of refractive cataract surgery [25]. Furthermore, since the IOLMaster700 was introduced in 2014, it became the first representative of a new family of optical biometers based on SS-OCT where it had an increased ability to evaluate advanced cataracts and measure LT very precisely, which was not possible with most optical biometers based on partial coherence interferometry [22].

As one of the important biometric parameters, LT not only plays an increasingly important role in the surgical planning of cataract patients [6, 26], but also correlates to morphological changes in the lens with aging or cataract formation [7, 8]. Based on a large sample of Chinese patients, we reported the distribution of LT in cataractous eyes with the mean value being 4.51 ± 0.46 mm. Previously, Klein et al. measured LT from slit lamp photographs and reported a mean value of 6.17 mm [20], which was apparently higher compared to the studies using ultrasound or optical biometry. However, for ultrasound biometry, the mean LT varied significantly from 3.95 mm to 4.94 mm in previous studies [27, 28]. Recently, with the advancement of optical biometry, LT can be measured precisely in cataractous eyes [22]. Our result was similar to those of the studies based on the Lenstar LS 900 (4.59 mm) and IOLMaster700 (4.56 mm) [10, 28]. Besides, the average LT of the Asian eyes in our study was similar to those of previous studies on cataract patients in North America and Southeast Asia, such as 4.38 mm in the Los Angeles Latino Eye Study [29], 4.51 mm in the Meiktila Eye Study [30], and 4.62–4.79 mm in the Tanjong Pagar Survey [31]. Moreover, LT was found to be significantly related to age in the study population. The increasing trend of LT with age, which is consistent with previous observations on human adults and animal models [27, 32], may result from the continuous accretion of lens fibers in the equatorial region of the lens [7]. Pertaining to gender, the correlation between a thicker lens and the male gender has also been reported by previous studies, such as the Beaver Dam Eye Study and the Central India Eye and Medical Study [20, 27], which may be paralleled by similar associations between AL and male gender [33].

LT, as a factor of the anterior segment anatomy, may be closely related to the biometric factors that further influence the estimation of postoperative position of IOL [11, 34], though it was not included in several previous IOL calculation formulas [35, 36]. Recently, new-generation formulas including the Olsen, Holladay 2 and Barrett Universal II formulas also rely on LT and have shown improved prediction accuracy [3–5]. For cataract surgeons today, we recommend LT to be introduced in IOL power calculation, and that new-generation formulas that are less influenced by LT [37], such as Barrett Universal II, Kane, PEARL-DGS, and EVO V2.0 may be applied. In this study, multiple risk factors for LT were identified, including older age, male gender, thicker central cornea, shallower ACD, larger WTW, and flatter anterior corneal curvature. For patients with these characteristics, surgeons need to know whether they have extreme LT before surgery, which may result in a greater hyperopic shift after surgery [19], and then proceed to use IOL formulas to gain a stable performance in a wide range of LT.

In our study, the association of LT with CCT agrees with previous reports [37], suggesting that the thicker lens may correspond with a thicker cornea. Moreover, we identified a negative correlation between LT and ACD. With the progression of cataract, the lens tends to thicken bi-directionally, which may result in a shallower anterior chamber [38]. Due to this correlation, IOL formula bias may be expected when the preoperative ACD is used alone rather than in combination with the LT, though the statistical correlation between the preoperative ACD and the postoperative position of the IOL has been widely proven [6]. Another interesting observation is the positive correlation between LT and WTW. In clinical practice, the WTW may provide helpful information for capsular bag sizes since it is difficult to directly measure capsular bag sizes due to the lack of convenient measurement devices. Large WTW may be associated with the development of larger anterior segments, which also indicates the incompatibility between fixed-size IOLs and large capsular bags, which may further affect IOL stability [39]. Thus, in cataract patients with a thicker lens, the IOL should be also chosen with caution. In addition, we also report the association between LT and cornea curvature, which may provide a clue to the role of LT in the anterior segment anatomy.

More importantly, our study revealed that the distribution of LT in dependence of AL was not simply linear. This trend can only be clearly revealed with such a large sample. Previously, some studies found greater LT was associated with shorter AL, but only with a narrow range of AL [27]. However, in our study, we found that with the elongation of AL, the LT firstly slightly increased to a maximum of 4.75 ± 0.45 mm in the 20.01–22 mm AL group, then gradually decreased to a minimum of 4.33 ± 0.45 mm in the 26.01–28 mm AL group, and finally increased again and reached a peak when AL was greater than 35 mm. As for short eyes, the correlation between LT and AL was positive but weak, possibly because the lens development could either be normal or abnormal in the early stages of these eyes [40]. Moreover, it seems that moderate myopic eyes tend to have thinner lenses than emmetropic eyes. The thinning of the lens in myopic eyes may be an indication of the lens trying to control overall refractive status towards emmetropia or achieve a clear image on the retina [41]. However, unlike the myopic eyes with AL ≤ 26 mm, the LT of highly myopic eyes increases with AL again, though future studies are needed to identify whether the increase of LT is related with the global expansion of highly myopic eyes or resulted from metabolic changes in the lens [42].

Furthermore, the nonlinear change of LT with AL may, to some extent, contribute to the prediction errors of the previous formulas that did not include LT [43]. The impact of LT on IOL calculation has been confirmed in recent studies. Hyperopic shift is associated with a thicker lens, while a myopic shift is associated with a thin lens, especially for the Haigis formula which did not include LT [19, 25, 44]. Even for formulas that included LT, the effect of LT on refractive error is varied with different ALs, with the greatest effect seen in eyes with AL less than 22 mm [18]. Besides, there is a positive correlation between LT and postoperative IOL position as demonstrated in previous studies [3, 26]. Thus, the nonlinear change of LT with AL may account for some bias in IOL power calculation for short or long eyes, and the regression models for predicting IOL position in normal eyes may not fit in these eyes with extreme ALs. Although the choice of IOL power formulas in short or long eyes has been given much attention, the contribution of LT to the bias of IOL power calculation is unrecognized. Therefore, our study suggests that LT may be a vital variable to be considered during IOL calculation in short or long eyes. Moreover, the individualized selection of IOL formulas and optimization of the formula coefficient or constant may be needed to reduce prediction bias.

## Conclusions

In conclusion, based on a large sample of Chinese cataractous population, a thicker lens was found to be associated with older age, the male gender, thicker central cornea, shallower ACD, larger WTW, and flatter anterior corneal curvature. In addition, the distribution of LT against AL is not simply linear, with the thickest lens seen in eyes with AL ranging 20.01–22 mm and thinnest lens in eyes with AL ranging 26.01–28 mm. Future study is needed to analyze the refractive prediction error of different IOL formulas using preoperative LT in eyes with very thick or thin lenses and short or long AL. The correlation of the predicted IOL position using age, AL, ACD, cornea curvature, and LT with postoperative lens position may also be needed.

## Data Availability

Available from the corresponding author on reasonable request.
